# Use of Rice Husk as a Biosorbent for Analytical Purposes: Physicochemical, Morphological Characterization and Application in Human Breast Milk

**DOI:** 10.1021/acsomega.4c11200

**Published:** 2025-06-06

**Authors:** Leonardo Corrêa Cardoso, Gustavo Andrade Ugalde, Tielle Moraes de Almeida, Jaquelini da Silva Reis, Maria Odete da Silva Dalan, Fernanda Ziegler Reginato, Berenice de Oliveira Cruz Rodrigues, Cristiane de Bona da Silva, Fábio Andrei Duarte, Ederson Rossi Abaide, Marcio Antonio Mazutti, André Valle de Bairros

**Affiliations:** † Núcleo Aplicado à Toxicologia (NAT), Departamento de Análises Clínicas e Toxicológicas, Centro de Ciências da Saúde, 28118 Universidade Federal de Santa Maria, Santa Maria 97105-900, Brazil; ‡ Programa de Pós-Graduação em Ciências Farmacêuticas, Centro de Ciências da Saúde, 28118 Universidade Federal de Santa Maria, Santa Maria 97105-900, Brazil; § Laboratório Integrado de Manejo de Pragas (LabMIP), Departamento de Defesa Fitossanitária, Centro de Ciências Rurais, Universidade Federal de Santa Maria, Santa Maria 97105-900, Brazil; ∥ Laboratório de Pesquisa em Nanotecnologia e Desenvolvimento Farmacotécnico (NDF+), Departamento de Farmácia Industrial, Centro de Ciências da Saúde, Universidade Federal de Santa Maria, Santa Maria 97105-900, Brazil; ⊥ Laboratório de Superfícies e Macromoléculas (SMLab), Departamento de Física, Centro de Ciências Naturais e Exatas, Universidade Federal de Santa Maria, Santa Maria 97105-900, Brazil; # Unidade Obstétrica, Hospital Universitário de Santa Maria, 28118 Universidade Federal de Santa Maria, Santa Maria 97105-900, Brazil; ∇ Programa de Pós-Graduação em Química, Departamento de Química, Centro de Ciências Naturais e Exatas, Universidade Federal de Santa Maria, Santa Maria 97105-900, Brazil; ○ Programa de Pós-Graduação em Engenharia Química, Centro de Tecnologia, 28118 Universidade Federal de Santa Maria, Santa Maria 97105-900, Brazil

## Abstract

The secretion of medicines in breast milk is a crucial topic in clinical medicine. LC-DAD stands out in the literature for its simplicity, cost-effectiveness, and reproducibility. Chromatographic techniques, often coupled with cleaning methods such as μQuEChERS, have proven efficient in analyzing drugs across biological matrices. This study aims to characterize alternative sustainable biosorbents with a high silica content for analytical use, aiming to reduce environmental impact. Commercial sorbents (PSA, C18, GCB) and biosorbents (rice husk (RH), cork, orange bagasse, and passion fruit) were evaluated, with RH presenting superior results. RH was then characterized through laser diffraction, SEM, FT-IR spectroscopy, X-ray diffraction, and thermogravimetric analysis, among others. Key results include SPAN (2.567 ± 0.09), *D*[4,3] (126 ± 27), and a nonuniform morphology compared to commercial sorbents. RH composition was 27% cellulose, 13% hemicellulose, 9% lignin, and 23% extractives, with notable properties such as WRI (72%) and ZP (−19.2 ± 0.8). Unlike prior studies, this work provides unprecedented physicochemical and morphological characterization of biosorbents, demonstrating RH as a promising and sustainable alternative to traditional sorbents like C18.

## Graphical abstract

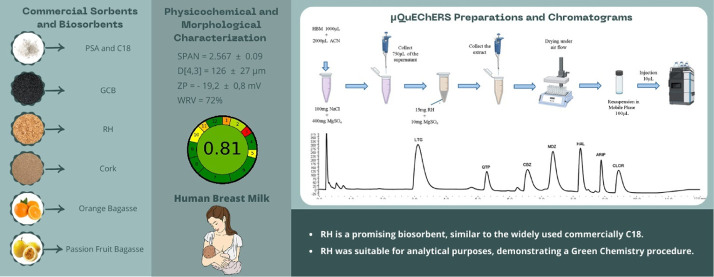

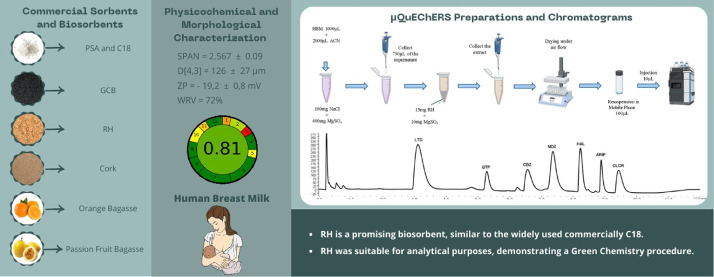

## 1 Introduction

Various industrial sectors have generated a significant amount of waste, the improper disposal of which can result in an environmental imbalance. In this context, the 2030 Agenda for Sustainable Development created by the United Nations Organization represents a global action plan composed of 17 sustainable development goals, which establish innovation through sustainable practices and the preservation of nature.^1^

Thus, it becomes essential to promote the use of renewable materials and advance the development of methodologies and technologies to produce goods, adding value to materials that would otherwise be discarded. Additionally, there is a notable persistence in the manufacture of products using raw materials harmful to the environment when sustainable alternatives could be adopted.^2–6^

In recent years, there has been an increase in the use of biosorbents for analytical purposes, mainly for the QuEChERS (*Quick, Easy, Cheap, Effective, Rugged, Safe*) procedure,^7^ in which conventional commercial sorbents have been replaced by waste materials such as sugarcane bagasse,^8,9^ pine bark,^10^ crab shell powder,^11^ chitosan,^12^ and cork.^13–15^ These studies used biosorbents in the extraction process of various heavy metals and xenobiotics (hormones, pesticides, antidepressants, and benzodiazepines) in nonbiological and biological matrices.^8–15^

In this sense, alternative residues from biomaterials can be used as biosorbents such as orange bagasse, passion fruit bagasse, cork, and rice husk (RH). RH has been demonstrated to be an interesting alternative as a biosorbent for the μQuEChERS procedure, considering global rice production and the disposal of RH, which generates tons of waste.^16^ Also, RH can be a potential substitute for industrial silica and a sustainable material rich in silica.^17,18^

So, this study aimed to characterize the physicochemical and morphological aspects of RH, comparing it with other possible alternative biosorbents (orange bagasse, passion fruit bagasse, and cork), as well as its application in real samples to replace commercial sorbents for analytical purposes.

## 2 Materials and Methods

### 2.1 Materials

Commercial sorbents superabsorbent polymer (PSA), carbon 18 (C18), and graphitized carbon black (GCB) were obtained from Sigma-Aldrich (St. Louis, USA). Orange and passion fruit bagasse and RH were obtained as waste materials from the agronomy department of UFSM, while cork was obtained from discarded wine corks. Acetonitrile and methanol solvents were obtained from Merck (Darmstadt, Germany), and the reagents magnesium sulfate, sodium chloride, and trifluoroacetic acid were obtained from Sigma-Aldrich (St. Louis, USA). Reference standards of carbamazepine (CBZ), lamotrigine (LTG), aripiprazole (ARIP), chlorpromazine (CLOR), haloperidol (HAL), quetiapine (QTP), and medazepam (MDZ) were obtained from US Pharmacopeia (USP).

Human breast milk (HBM) samples were obtained from volunteers at the University Hospital of Santa Maria. An aliquot of 5 mL of HBM was sufficient for the analytical procedures. These samples were stored in appropriate containers identified only by numbers to preserve patient identity and stored in a −80 °C freezer. The volunteers filled out informed consent forms for the donation of HBM. This study was approved by the Human Ethics Committee of the Federal University of Santa Maria (CAAE: 58225622.3.0000.5346; No. 5.422.164).

### 2.2 Chromatographic Method and Sample Preparation

To select the best commercial sorbent and, subsequently, the appropriate biosorbent, a method of liquid chromatography with a diode array detector (LC-DAD) was developed. Experiments were conducted on a Nexera-XR system (Shimadzu, Kyoto, Japan) equipped with an LC-20ADXR model pump, an SPD-M20A UV/vis detector, a SIL-20AXR (20 μL injection volume) autosampler model, a column oven model CTO-20A, and a system controller CBM-20A, with the following specifications: mobile phase: H_2_O + 0.1% trifluoroacetic acid (A):methanol (B); column: Agilent Eclipse Plus C18–3.5 μm 2.16 × 50 mm (Santa Clara, USA); flow rate: 0.7 mL/min; wavelength: 220 nm; injection volume: 10 μL. The gradient system between the percentages of moving phases A and B used was as follows: 0–4 min (80:20=A:B), 4–5 min (65:35=A:B), 5–7 min (65:35=A:B), 7–8 min (50:50=A:B), 8–10 min (50:50=A:B), 10–11 min (80:20=A:B), and 11–12 min (80:20=A:B).

The method detects the anticonvulsants CBZ and MDZ (internal standard: IS), LTG, and the antipsychotics ARIP, CLOR, HAL, and QTP in HBM (LoD: 500 ng/mL). All HBM samples were fortified with 10 μg/mL of the above analytes. The sample preparation was performed according to Figure 1.

**Figure 1:**
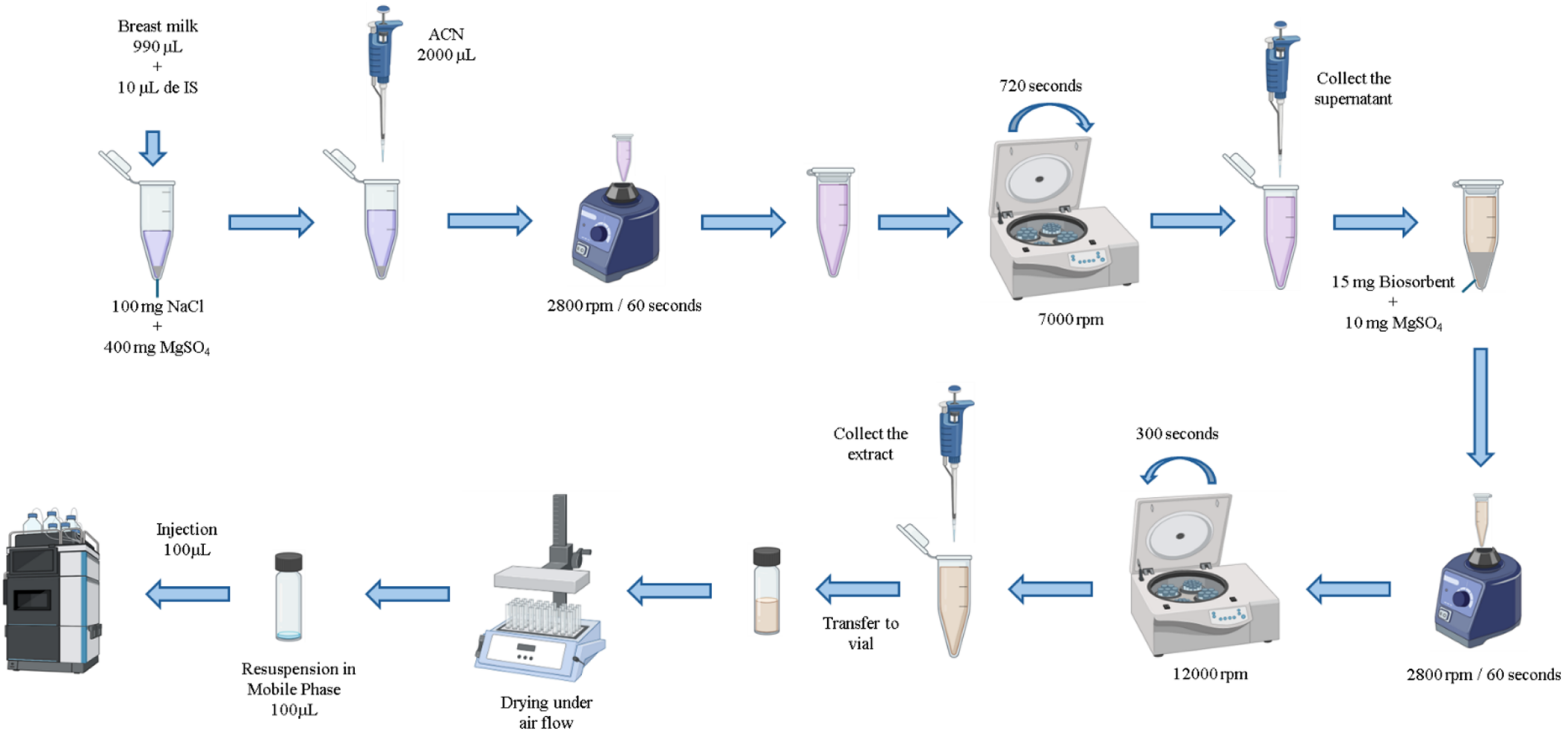
Sample preparation was carried out by μQuEChERS.

### 2.3 Screening of Commercial Sorbent

To determine the best commercial adsorbent, its use was established in the cleaning phase using the QuEChERS technique described by Anastassiades et al.,^7^ adapted for a miniaturized version. The samples were evaluated according to the chromatographic method previously developed for this study.

### 2.4 Screening of Biosorbent

To determine the biosorbent, the commercial adsorbent was replaced by the natural one in the cleaning phase in the μQuEChERS technique. The samples were also evaluated using the same chromatographic method and compared with the best commercial sorbents previously defined.

### 2.5 Particle Size Polydispersity, Zeta Potential (ZP), Electrophoretic Mobility, and Conductivity Analysis

The Mastersizer 3000 (Malvern Instruments, Bristol, UK) instrument determined the granulometric distribution profile. Samples were added directly into the equipment’s disperser compartment containing 250 mL of distilled water until the appropriate obscuration index (10–12%) was achieved. The refractive index for cellulose derivatives (1.470) was applied in the analysis.^19^ For each sample batch, the volume-weighted mean diameter in μm (*D*[4,3]) and the polydispersity index of the formulations (SPAN) were evaluated. ZP, conductivity, and electrophoretic mobility were determined by laser Doppler microelectrophoresis at 25 °C using the zetasizer. For this, 100 mg of the biosorbent was weighed and diluted 500 times in a 10 mM NaCl solution to ensure proper dispersion and accurate measurement.

### 2.6 Scanning Electron Microscopy (SEM)

Morphological analysis was performed using a scanning electron microscope (TM3030, HITACHI, Japan) at an acceleration voltage of 15 kV. For the analysis, the samples were coated with a thin layer of gold, and their shape and surface characteristics were analyzed at various magnifications.

### 2.7 Ash, Humidity, and Total Extractives Determination

Ash, humidity, and total extractive determinations were performed directly on sorbent samples according to the methodologies recommended by the Association of Official Analytical Chemists and the National Renewable Energy Laboratory^20^ in three independent experiments, where the results are reported as means ± standard deviation.

### 2.8 Determination of Cellulose, Hemicellulose, and Lignin

Methodology recommended by the National Renewable Energy Laboratory was also used to determine cellulose, hemicellulose, and lignin.^20^ A quantity of 0.3 g of pretreated sorbent was individually weighed. The acid hydrolysis step was performed later, and a portion of the resulting solution was analyzed using a UV–visible spectrophotometer (BEL, model UV-M51, Italy) to measure the absorbance and determine the soluble lignin content.

The remaining hydrolysis solution was used to determine the cellulose and hemicellulose. After filtration on cellulose filter paper, the sample was rinsed with ultrapure water. Subsequently, it was dried in an oven (Lucadema, model LUCA 80/27, Brazil) at a constant temperature of 105 °C until a constant weight was reached for the determination of insoluble lignin. All determinations were performed in quadruplicate, and the results were presented as means ± standard deviation.

### 2.9 Thermogravimetric Analysis (TGA)

Thermogravimetric analysis of fresh sorbents was performed on a thermogravimetric analyzer (DTG60/60H, Shimadzu, Kyoto, Japan). The analysis involved heating 20 mg of the sample at a rate of 10 °C/min from room temperature (25 °C) to 850 °C in a nonoxidizing atmosphere (N_2_, 99.997% purity) at 100 mL/min. The thermogravimetric data were converted into derivative thermograms (DTG).

### 2.10 Fourier Transform Infrared Spectroscopy (FT-IR)

Pretreated RH samples were analyzed by FT-IR spectroscopy (IR Prestige 21, Shimadzu, Kyoto, Japan). The spectrum was obtained with a nominal resolution of 4 cm^–1^ within the spectral range of 4000–400 cm^–1^. All discs were made in the potassium bromide (KBr) pellet matrix accessory using a press (Manual Press SSP-10, Shimadzu, Kyoto, Japan). For disc preparation, 100 mg of KBr and 1 mg of the sample were used, thoroughly ground using a mortar and pestle, and then pressed under about 8 tons, resulting in thin, transparent discs with less than 1 mm thickness and 13 mm diameter.

### 2.11 X-ray Diffraction (XRD)

Crystalline index and identification of silica peaks were performed using an X-ray diffractometer (Rigaku, MiniFlex 300, Japan) equipped with copper Kα radiation (1.54051 Å) and 2θ = 5–60°, with 1° divergence and reception slits at 30 kV and 10 mA.

### 2.12 Water Retention Index (WRI)

The WRI of the biosorbent was determined according to Pakutsah and Aht-Ong.^21^ A sample equivalent to 5 g of dry biosorbent, previously hydrated for 24 h, was centrifuged under standard conditions (relative centrifugal force—RCF = 900 G, time = 30 min, and temperature = 21 ± 3 °C) and then transferred to an oven at 105 ± 3 °C until a constant mass was achieved. The masses of the biosorbent before (wet mass, WM) and after drying in the oven (dry mass, DM) were determined, and the WRI of the gel was calculated by using the equation below. This analysis was performed with five repetitions for each biosorbent sample.

$$\mathrm{WRI}=\left(\frac{\mathrm{WM}-\mathrm{DM}}{\mathrm{DM}}\right)\times 100$$

### 2.13 Statistical Evaluation

The statistical methodology applied in this study was based on each analyte and different sorbents. Commercial sorbents such as PSA, C18, and GCB were initially compared to evaluate their adsorption capacity. Based on these findings, natural sorbents including rice husk, cork, orange bagasse, and passion fruit were tested directly with the best commercial sorbent. The samples of each sorbent were analyzed in triplicate, and the areas of the chromatographic peaks were used as indicators of adsorption efficiency. The normality of the data was evaluated by the Shapiro–Wilk test.

To evaluate the adsorption capacity of several sorbents, a comparative analysis was performed on six types of sorbents: PSA, C18, RH, cork, orange, and passion fruit bagasse. The dependent variable in this study was the percentage adsorption area, with PSA serving as a reference sorbent. After the normality of the data was confirmed by the Shapiro–Wilk test, the data set was submitted for analysis of variance (ANOVA) to detect general differences between the sorbents. Subsequently, a post hoc Tukey test was applied to identify specific differences by pair, ensuring statistical accuracy in distinguishing significant variations between sorbents. Statistical analysis was performed with Statistica 12.0, with a significance level (α) defined at 0.05. This method allowed the precise identification of which sorbents showed significantly different adsorption performances about PSA and C18, based on the magnitude of their percentage areas. For particle size analysis (*D*[4,3]) and its homogeneity (SPAN), a one-way ANOVA test was performed with Tukey’s test as post hoc. GraphPad Prism version 8.01 (GraphPad Software, Inc.) was used for graphs and statistical analysis for the particle size.

## 3 Results

### 3.1 Screening of Commercial Sorbents

Preliminary tests were conducted in triplicate with commercially available sorbents commonly used in the μQuEChERS technique (PSA, C18, and GCB) to evaluate the adsorption power of the sorbents and select the best for the cleaning phase of the technique in HBM samples. GCB is not represented in Figure 2 due to the high relative standard deviation (>60%), which did not allow for an appropriate evaluation of this material for analytical purposes based on HBM samples. The mean absolute area obtained with experiments for PSA, the most used in commercial kits of the technique, was considered 100% for better visualization of the results according to Figure 2.

**Figure 2:**
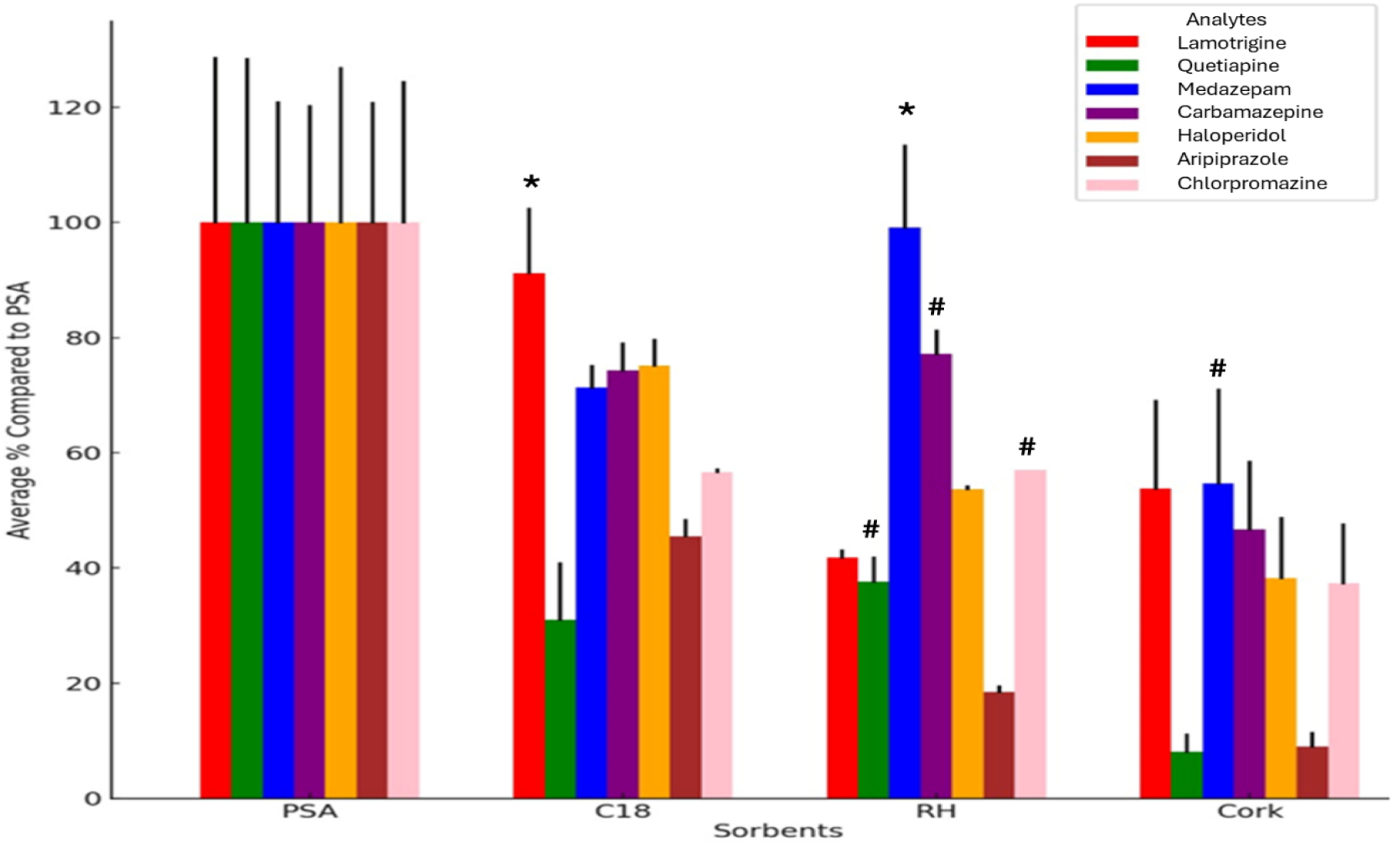
Comparison between commercial and biosorbent in area percent considering PSA as 100%. *Not a significant difference with PSA (*p* < 0.05) for ANOVA with Tukey’s test post hoc; # not a significant difference with C18 (*p* < 0.05) for ANOVA with Tukey’s test post hoc; PSA—superabsorbent polymer; C18—carbon 18 (*octadecyl silyl)*; RH—rice husk; GCB is not represented in the figure due to high RSD (>60%).

### 3.2 Screening of Natural Sorbents

Preliminary tests were conducted in triplicate with natural sorbents suggested as possible biosorbents in the μQuEChERS methodology (RH, orange bagasse, passion fruit bagasse, and cork) and compared to the best commercial sorbent. Subsequently, evaluation of normality using the Shapiro–Wilk test revealed that most data sets deviated from normality (*p* < 0.05).

Statistical analysis using ANOVA with a post hoc test of Tukey revealed different adsorption behaviors between natural sorbents when compared to traditional C18 and PSA sorbents. Notably, PSA exhibited a significantly higher adsorption efficiency than C18, as indicated by a *p*-value of 0.022386. Compared to C18, biosorbents such as RH and cork did not show significant statistical differences for most analytes tested, suggesting their performance was interchangeable with that of a commercial sorbent.

### 3.3 Particle Size Polydispersity Analysis

During polydispersity analysis, four different types of RH pretreatments were analyzed to optimize the biosorbent processing, evaluating particle size (*D*[4,3]) and its homogeneity (SPAN). The different pretreatments were:

A) Grinding in knife mill + ball mill with prior cleaning.
B) Grinding in knife mill + ball mill without prior cleaning.
C) Grinding in a ball mill with prior cleaning.
D) Grinding in a ball mill without prior cleaning.

Prior cleaning consists of washing the natural sorbent to remove exogenous substances, as reported by Ossanes et al.^15^ Through this preliminary analysis, it was possible to determine the best pretreatment, which was, according to Table 1, direct grinding in a ball mill with prior cleaning with five times sonicate in methanol for 30 min, until the RH becomes pale and then drying in a kiln at 70 °C overnight.^15^

**Table 1:** Physicochemical Characterization of RH^a^

Polydispersity of Particle Size		
Sample	SPAN	*D*[4,3] (μM)
A	2.393 ± 0.21	275 ± 20
B	2.417 ± 0.16	326 ± 20
C	2.567 ± 0.09	126 ± 27^b^
D	3.193 ± 0.28^b^	227 ± 10

Components (%, dry base)						
Biosorbent	Cellulose	Hemicellulose	Lignin	Extractives	Ash	Humidity
RH	26.83 ± 0.69	13.10 ± 0.52	9.07 ± 3.17	23.00 ± 1.60	19.82 ± 0.06	8.18 ± 0.28

WRI		
Biosorbent	Average WRI (%)	RSD (%)
RH	71.8	0.92

Zeta Potential, Electrophoretic Mobility and Conductivity Particle			
Sample	ZP (mV)	Electrophoretic Mobility (μm cm/(V s))	Conductivity (mS/cm)
RH	–19.7 ± 0.64	–1.547 ± 0.05	1.36 ± 0.06
	–18.9 ± 0.82	–1.479 ± 0.06	1.37 ± 0.05
	–18.9 ± 0.93	–1.479 ± 0.07	1.37 ± 0.05

a Rice husk (RH); relative standard deviation (RSD); water retention index (WRI); zeta potential (ZP).

b Significant value (*p* < 0.05) for ANOVA test with Tukey’s test as post hoc.

This pretreatment was used for the selection and for the other analyses of biosorbent characterization.

### 3.4 Determination of Zeta Potential (ZP), Electrophoretic Mobility, and Conductivity

ZP, electrophoretic mobility, and conductivity were evaluated through laser Doppler microelectrophoresis of suspended particles, as shown in Table 1.

### 3.5 Physicochemical Composition of RH and Thermogravimetric Analysis

The chemical composition of RH used in this study is presented in Table 1. The individual contributions of the components were determined by integration and normalized, as illustrated in Figure 3A,B.

**Figure 3:**
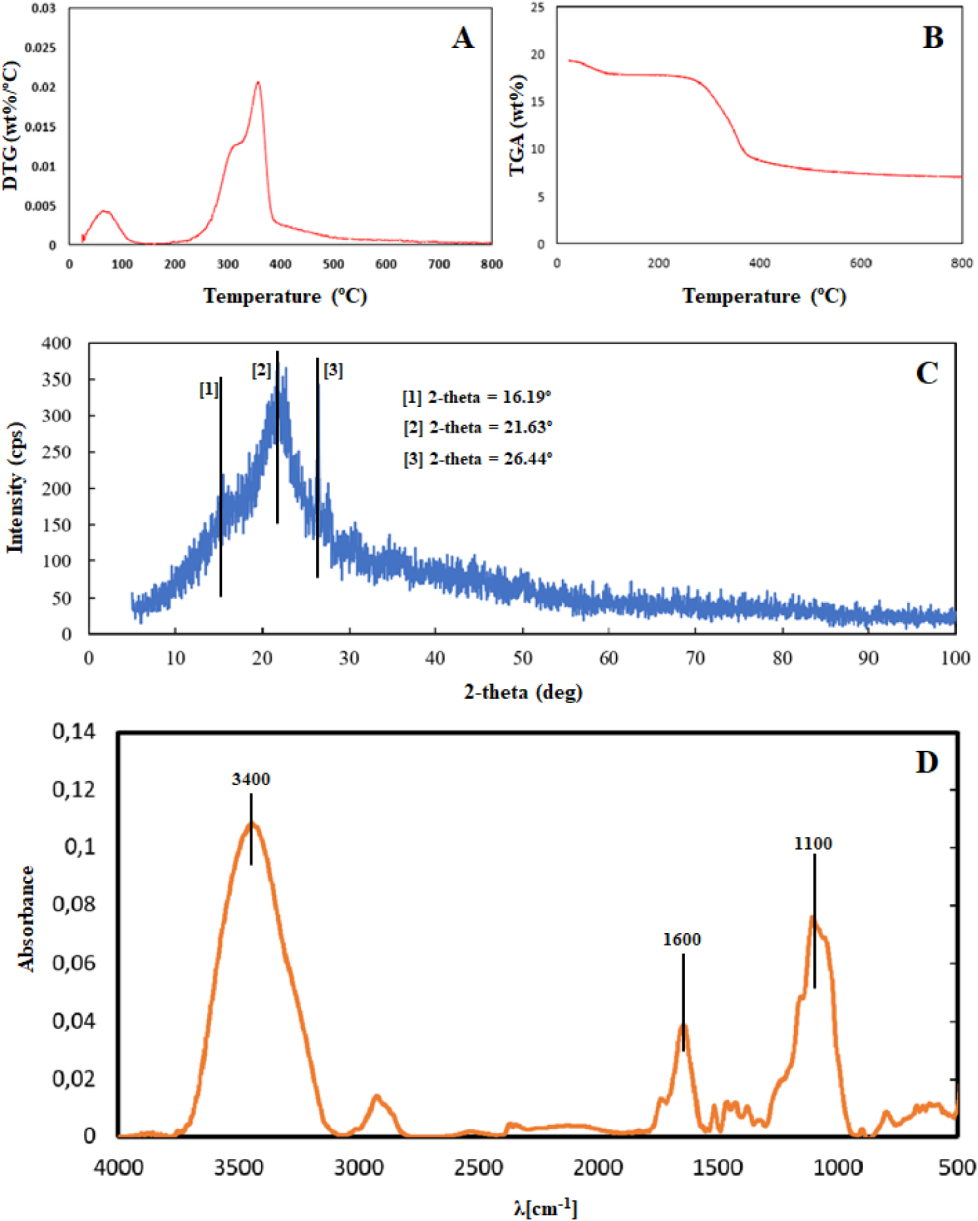
Physicochemical characterization of the rice husk. (A) TGA; (B) DTG; (C) X-ray powder diffractograms; (D) Fourier transform infrared spectroscopy (FT-IR) spectrum.

### 3.6 Fourier Transform Infrared Spectroscopy (FT-IR) and X-ray Diffraction (XRD)

Figure 3C,D shows the data from the FT-IR and XRD, respectively. Figure 3C shows a wide peak between 20 and 30% (2θ) with a maximum intensity of 2θ = 22.28. In parallel, the figure also reveals an acute peak with maximum intensity observed at 2θ = 26.48%.

### 3.7 Scanning Electron Microscopy (SEM)

Analysis of the material by SEM revealed that the surface of RH presented a morphology consistent with native cell walls with compact surface structures of intact morphology. The RH surface has a grain fracture, which may have occurred due to the grinding step in the biosorbent pretreatment before characterization tests, as shown in Figure 4.

**Figure 4:**
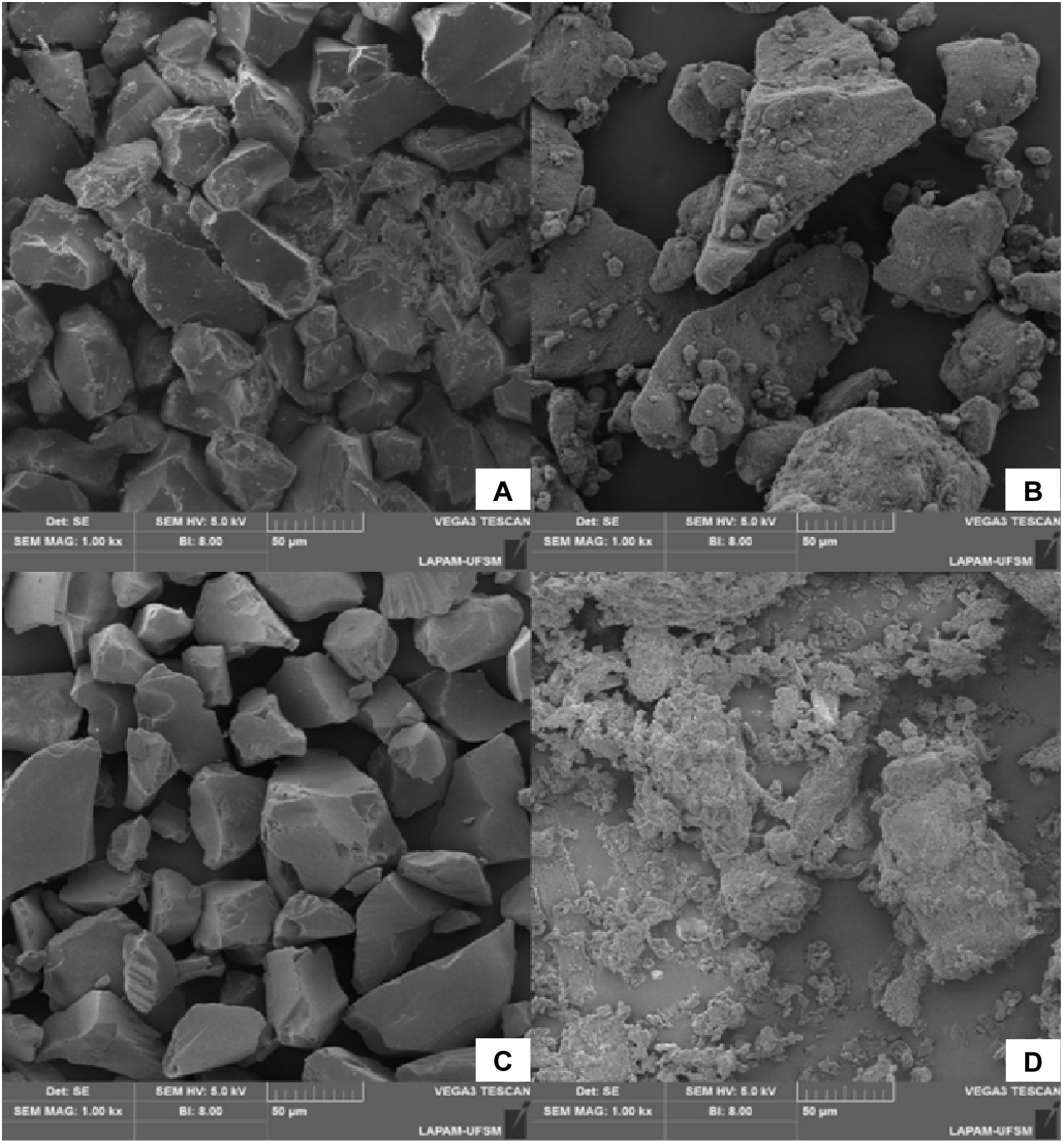
SEM images of commercial sorbents and RH samples (1000×). (A) C_18_; (B) GCB; (C) PSA; (D) RH.

## 4 Discussion

This study addresses a crucial gap in the literature, which was previously ignored in the selection and characterization of natural products for analytical purposes. Thus, the research begins with a screening of the main sorbents found in commercial kits available on the market, where PSA was the best among them for the cleaning phase of μQuEChERS for analysis of anticonvulsants and antipsychotics in HBM. Subsequently, biosorbents (RH, cork, orange, and passion fruit bagasse) were compared to PSA for adsorbent capacity, as shown in Figure 2. During the analysis of the chromatograms of sorbents and biosorbents (Supporting Information), orange and passion fruit were excluded from the study due to the high amount of interference of the biosorbents themselves and the difficulty in reproducibility among extractions.

Due to the statistical analysis performed (Figure 2), the PSA sorbent was the best option for the cleaning phase in the μQuEChERS technique. However, RH and cork did not demonstrate different statistics compared to the C18 sorbent (used in commercial kits), demonstrating the enormous potential of these biosorbents for analytical proposals. RH has a high silica content, which is likely involved in specific interactions with pharmaceutical compounds, possibly through the formation of hydrogen bonds or electrostatic interactions with the silica surface.^22^ Considering the cork constitution (mainly of suberin and lignin), this biosorbent offers a unique porous structure that facilitates the retention of hydrophobic and slightly polar compounds.^23^

However, the statistical results demonstrate the superiority of RH in relation to cork, since it presents greater adsorption efficiency for almost all analytes (except LTG) when submitted to μQuEChERS. The greater presence of hemicellulose and cellulose in RH compared to cork (Table 1) results in a higher RH hemicellulose–cellulose/lignin ratio, possibly influencing the extractive capacity of the analyzed substances. Likewise, RH was the only sorbent that showed MDZ with results analogous to those of PSA. Subsequently, an extraction based on μQuEChERS using RH was performed and compared to that of PSA to demonstrate analytical feasibility for samples of HBM, as shown in Figure 5.

**Figure 5:**
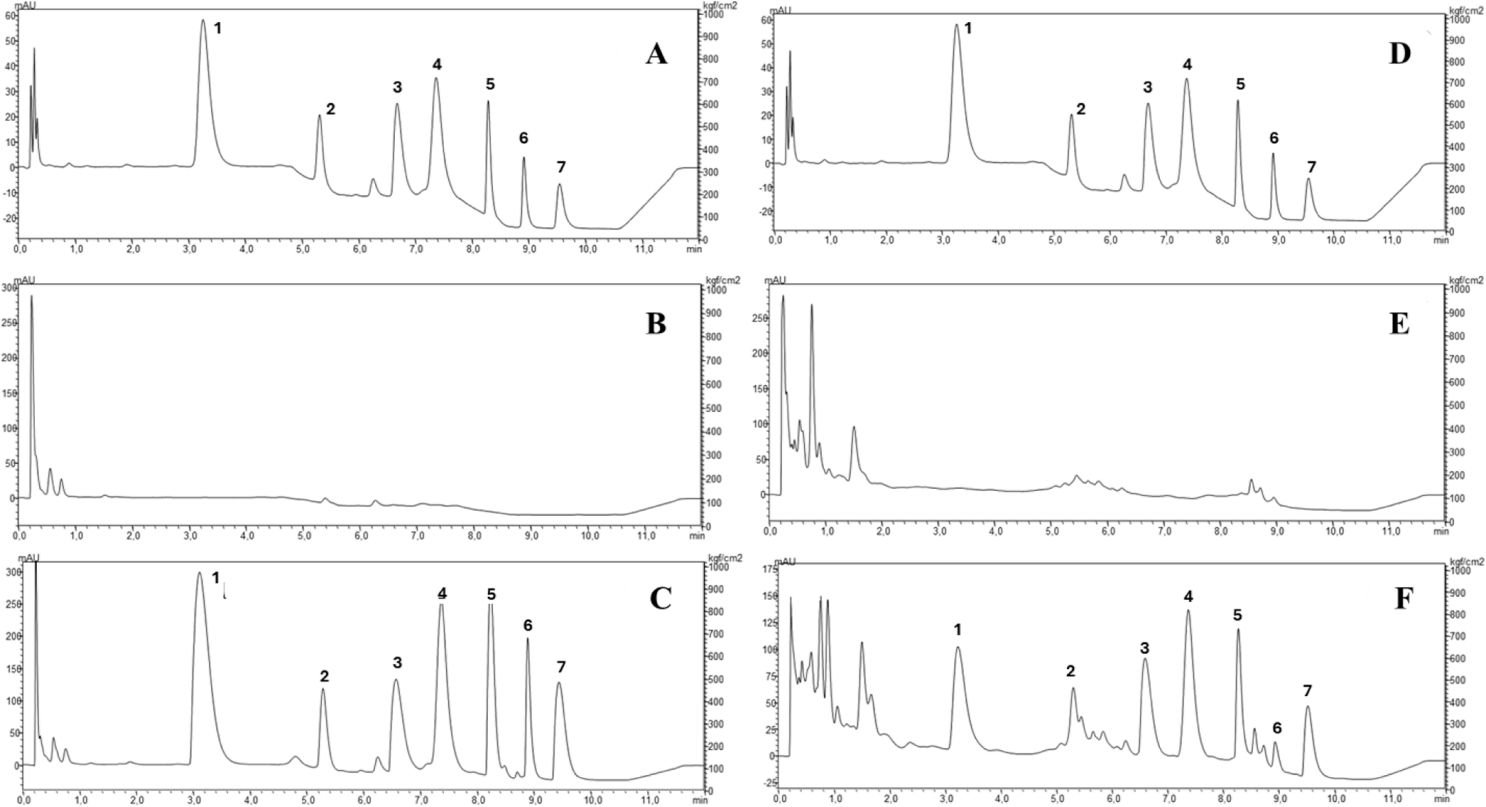
μQuEChERS Chromatograms: PSA vs RH. (A) Standards PSA; (B) blank PSA; (C) sample PSA; (D) standards RH; (E) blank RH; (F) sample RH; 1—LTG; 2—QTF; 3—MDZ (IS); 4—CBZ; 5—HAL; 6—ARIP; 7—CLOR.

Based on particle size analysis, it was determined that the best pretreatment for the continuation of the physicochemical and morphological characterization of RH was direct grinding in a ball mill with prior cleaning with five times sonicate in methanol for 30 min, until the RH becomes pale and then drying in a kiln at 70 °C overnight (Table 1), corroborating with the results obtained by Birk et al.^14^ and Ossanes et al.^15^ In addition, the direct grinding results showed significant results in particle size and a slightly elevated SPAN (above 2.00), which ratifies the nonhomogeneity of the particles,^24,25^ increasing its adsorption capacity and demonstrating the promising use of RH as a biosorbent for μQuEChERS. However, this is the first study to use particle size and its homogeneity to characterize a biosorbent for analytical purposes.

According to Figure 4, RH reveals an uneven pattern in its shape compared with commercial sorbents such as C18, PSA, and GCB at 1000× magnification. This unevenness in particle shape is maintained at an increase of 5000× (Figure S1) and corroborates with the data reported in the study by Bhatia and Sahu.^26^ The presence of pores in the particle was not visible even at the highest increase in SEM. The Brunauer, Emmett, and Teller surface area test (BET) would be an option for this evaluation;^13,17^ however, we do not have access to such a technique. Nevertheless, this irregularity in the shape of RH particles tends to increase the adsorption efficiency in the sample cleaning phase by providing chromatograms with less residual biosorbent artifacts, which does not occur with orange and passion fruit bagasse (Figure S2).

The values obtained for cellulose, hemicellulose, lignin, ash, and humidity (Table 1) were close to those found by Abaide et al.,^27^ although it is difficult to make direct comparisons of chemical compositions depending on the type and conditions of cultivation.^26–28^ Regarding the ash content, it is possible to observe that this content in RH (19.82 ± 0.06) is relatively high compared to other types of biomasses, such as soybean husks, which in the study conducted by Vedovatto et al.,^28^ had an ash content of 4.65 ± 0.29. This result can be attributed to the high silica content present in RH, which varies between 15% and 17%, highlighting that most of the ash content of RH is composed of silica, as described by Marin et al.^29^

Extractive content, low molecular weight organic compounds such as phenolic compounds, terpenes, saturated and unsaturated fatty acids, proteins, and flavonoids, found in RH was higher than that found by Fleig et al.^30^ (11.0 ± 0.3%). This difference may have occurred due to the time of grain harvest and the region being different for this study.^31^

RH was submitted to TGA analysis to determine the thermal degradation of the material (Figure 3A). Analysis of thermogravimetric derivatives (DTG) was employed according to the methods recommended in the literature^32,33^ to determine the mass composition of hemicellulose, cellulose, lignin, and coal (Figure 3B and Table 1). TGA and DTA analysis showed that the silica contained in RH can absorb water and other substrates such as xenobiotics already demonstrated by Abaide et al.^27^ and Adenuga et al.,^34^ ratifying the use of RH as a biosorbent.

The XRD analysis revealed a broad peak between 20° and 30° (2θ) with a maximum intensity at 2θ = 22.28°, consistent with the characteristics of amorphous silica (SiO_2_).^35,36^ Additionally, a sharp peak with maximum intensity at 2θ = 26.48° was observed, which also indicates the presence of SiO_2_ polymorphs.^37,38^ Supporting these findings, studies of cellulose exhibit very similar patterns using the same technique. Cellulose presents peaks of significant intensity at approximately 22° and 16°.^39–41^ While the former is attributed to the crystalline structure, the latter reflects cellulose’s amorphous regions or polymorphism.^39–41^

According to the FT-IR spectrum for RH (Figure 3D), the peak in the region of approximately 3400 cm^–1^ can be attributed to the vibration of the O–H bond, which may correspond to lignin, as well as it can be attributed to the vibration of the SiO–H bond, H–O–H of water physically adsorbed in the material, and finally to the vibration of the Si–OH bond of the silanol groups present in the material.^42–44^ The peak obtained in the region of approximately 1100 cm^–1^ can be attributed to the vibration of C–O, C=C, and C–C–O bonds, referring to the cellulose polymer.^33^ Furthermore, Porrang et al.^45^ reported that the peak in the region of approximately 1100 cm^–1^ can be attributed to the asymmetric vibration of Si–O–Si bonds. Bands in the region of 1600 cm^–1^ represent the elongation of −OH present in silica structures in silanol (Si–OH) groups,^26,46–48^ as well as they may refer to the functional group C=O, which may be associated with ketone and aldehyde groups linked to the lignin of the untreated material.^42^ Thus, the promising results demonstrated in Table 1 and Figure 3A–D corroborate the use of RH as a natural sorbent for analytical proposals.

WRI of RH was evaluated, as shown in Table 1, obtaining a WRI of 71.8%, demonstrating that it has a high capacity to retain the aqueous phase in the cleanup phase of the μQuEChERS methodology. Furthermore, this result corroborates with literature findings for untreated rice, as reported by Kolya and Kang,^49^ who obtained a WRI of approximately 89%, considering the rice grain as a whole and not just the husk.

High silica content in RH plays a crucial role in the adsorption mechanisms of various matrix interferences present in the HBM composition, such as lipids, fatty acids, and sugars, among others.^27^ Studies indicate that the proportion of silica (SiO_2_) in RH ash is approximately 60%, while between 10% and 40% correspond to carbon derivatives and other minerals in smaller quantities.^35^ Moreover, RH is easily processed and regenerated, nonsensitive to toxins, and performs efficiently in environmental applications, with silica and carbon in its composition being the main components responsible for the adsorption mechanisms.^50,51^ Consistent with the literature,^50–55^ our research reaffirms the widespread use of RH as an effective biosorbent.

Moreover, the adsorption performance of natural sorbents from renewable agricultural sources, including fruit bagasse and RH, among others, can improve after chemical and thermal treatment. After heating the materials at high temperatures, several changes in the morphology of the natural form of the materials can occur due to the transformation of aliphatic carbon structures into aromatic carbon structures. Also, the porosity of these materials can be increased with chemical treatments, especially when potassium hydroxide (KOH) used.^34^

Draszewski et al.^56^ used subcritical water to hydrolyze RH at different temperatures during the hydrolysis process to obtain silica. For RH, the percentage of silica present in the ashes was 57.6%, and the total amount of silica in RH was 10.60 ± 0.6%, confirming the high proportion of silica present in RH, justifying its application as a potential biosorbent to be used in the cleanup step of the μQuEChERS methodology.

Furthermore, organic molecules previously present in untreated materials, which may interfere with adsorption and analytical performance after the development of the adsorption technique, are decomposed by thermal treatment.^34^ Thus, in the case of RH, which has a high silica content in its composition, the thermal decomposition of organic substances in the material increases the relative silica content. The increase in the weight percentage of silica positively influences the adsorption capacity of thermally treated RH due to the good adsorbent properties of silica.^57^

ZP is a key physical property that influences electrostatic interactions in particle dispersions, making it crucial for understanding the stability of colloidal systems.^58^ It is defined as the difference in potential between a particle’s surface and its surrounding ionic atmosphere, measured at the shear plane. Generally, a higher absolute value of ZP indicates better dispersion stability, with values above 30 mV considered ideal for maintaining stable conditions. In contrast, a ZP below 30 mV suggests a tendency toward aggregation, instability, flocculation, or coagulation.^59^

In pharmaceutical sciences, ZP is widely used alongside particle size to assess the stability and quality of micro- or nanoparticulate dispersions.^25,60–62^ Despite its common application in nanoparticle characterization, there are limited studies of using ZP for biomaterials. To date, no research has evaluated the ZP of RH particles. However, Li et al.^63^ analyzed ZP from rice flour and found a value of −13.52 mV, which is higher than this study’s average value of −19.2 mV. Our RH particles exhibit a negative ZP, negative mobility (−1.391 μm cm/(V s)), and good conductivity (1.37 mS/cm), indicating a strong potential for electrostatic interactions, which enhances the ability to capture charged molecules and improve the cleaning of biological matrices (Table 1).

The morphological and physicochemical characterization of RH particles was innovative because it is the first time that a polydispersion of particle size by light ray diffraction has been reported as well as its electrophoretic mobility and conductivity, which demonstrates the great relevance of this study of biomaterials characterization. Previously, the granulometric distribution was performed according to Table 2 by Almendros et al.^80^ and Grefa et al.^64^ However, several drum sieves of different sizes were used for the evaluation, and nonprecision equipment such as Mastersizer 3000 and ZP were only evaluated in the characterization of coconut husk for application in the removal of rhodamine 6G of wastewater.^88^

**Table 2:** Studies Published with Physicochemical and/or Morphological Characterization of Biosorbents in Several Matrices and Purposes^a^

					Morphological Characterization			Physicochemical Characterization													
					PDI			Components													
Biosorbent	Reference	LoD (ng/mL)	Sample (Matrix)	Analytical Methods	SPAN	*D*[4,3]	SEM	Cellu	Hemi	Lig	Ext	Ash	Hum	BET	WRI	ZP	Elet. Mob	Cond	TGA/DTG	XRD	FT-IR
Rice Husks (RH)	This work purpose	500	HBM	LC-DAD	X	X	X	X	X	X	X	X	X		X	X	X	X	X	X	X
	^17^	NI	NI	NI			X				X	X	X	X						X	X
	^27^	NI	NI	NI			X	X	X	X	X	X	X						X		X
	^64^	NI	Water	NI		*															
	^6^	NI	MSp	NI																	
	^65^	NI	NI	NI			X													X	X
	^66^	NI	NI	NI																X	X
Chitosan	^67^	NI	NI	NI																	
	^12^	100–5000	Rice	LC-MS/MS																	
Cork	^14^	0.5–5 (LoQ)	PB	LC-MS/MS			X	X	X	X	X	X	X								X
	^13^	1.5–3	Urine	LC-DAD			X							X							X
	^68^	3–19	Water	GC-MS			X	X	X	X	X	X	X								X
	^69^	0.004–0.03	RW	GC-MS			X	X	X	X	X	X	X								X
	^15^	5 (LoQ)	VH	LC–MS/MS			X	X	X	X	X	X	X								X
	^70^	0.24	Water	LC–MS/MS			X														X
	^71^	0.03	Water	GC-MS			X														X
	^72^	1–10	Water	GC-ECD				X	X	X	X	X	X								
	^73^	0.22	Urine	GC-FID			X												X		X
Orange Bagasse	^74^	95	WW	UV–vis																	
	^75^	NI	NI	NI			X	X	X	X	X	X	X						X	X	X
Passion Fruit Bagasse	^76^	NI	NI	NI			X													X	X
	^77^	NI	NI	NI			X							X					X	X	X
Sugarcane Bagasse	^8^	0.32–1.5	IE	LC–MS/MS																	
	^9^	0.43–0.79	Water	LC-MS/MS																	
Pine’s Products	^10^		LL	ICP-MS																	
	^78^	0.003–0.03	RW	GC-MS			X	X	X	X	X	X	X						X		
	^79^	0.03–3.03	Urine	LC-FLD			X														X
	^80^	NI	NI	NI		*	X	X	X	X	X	X	X	X					X		X
Sisal Fiber	^81^	3.7	Water	MP-AES																	
Activated Charcoal	^71^	NI	NI	NI																	
	^82^	NI	NI	NI			X													X	X
Walnut Shell	^83^	NI	WW	NI			X													X	X
Banana Peel	^75^	NI	NI	NI			X	X	X	X	X	X	X						X	X	X
Moringa Husks	^84^	0.75	GAS	FAAS																	
Brewer Spent Grain	^85^	50–200 (LoQ)	Urine	GC-MS-QP			X							X					X		X
Shrimp Co-products	^86^	NI	Wastewater	NI			X													X	X
Coconut Shell	^87^	NI	Wastewater	NI			X							X		X				X	X

a Limit of detection (LoD); limit of quantification (LoQ); flame atomic absorption spectrometry (FAAS); gas chromatography with electron capture detector (GC-ECD); gas chromatography with flame ionization detector (GC-FID); gas chromatography coupled with mass spectrometry (GC-MS); gas chromatography coupled with mass spectrometry single quadrupole (GC-MS-QP); inductively coupled plasma mass spectrometry (ICP-MS); liquid chromatography with diode array detector (LC-DAD); liquid chromatography with fluorescence detector (LC-FLD); liquid chromatography coupled with mass spectrometry (LC-MS); liquid chromatography coupled to sequential mass spectrometry (LC-MS/MS); liquid chromatography with ultraviolet detection (LC-UV); ultraviolet–visible spectroscopy (UV–vis); scanning electron microscopy (SEM); volume-weighted mean diameter (*D*[4,3]); Brunauer, Emmett, and Teller surface area test (BET); water retention index (WRI); zeta potential (ZP); thermogravimetric analysis/derivative thermograms (TGA/DTG); X-ray diffraction (XRD); Fourier transform infrared spectroscopy (FT-IR); electrophoretic mobility (Elet. Mob); conductivity (Cond); extractives (Ext); lignin (Lig); hemicellulose (Hemi); cellulose (Cellu); polydispersity index (PDI); human breast milk (HBM); microspheres (MSp); postmortem blood (PB); river water (RW); vitreous humor (VH); wastewater (WW); industrial effluent (IE); landfill leachates (LL); gasoline (GAS); *sieves of various sizes and not by laser diffraction; NI (not Informed).

Similarly, Table 2 shows the multiple gaps in the process of characterization of biomaterials, whether these biosorbents are used for analytical or industrial purposes. Thus, this study proves that a complete physicochemical and morphological characterization of RH certifies its best application only later.

In addition, regardless of this study focusing on the use of RH in its untreated form, further research should consider the application of high-temperature bioadsorbents chemically and thermally treated. These studies aim to expand the use of biodegradable and sustainable resources as viable alternatives in the cleanup step for μQuEChERS methodology to alternative matrices.

Furthermore, to confirm the potential of the green chemistry technique, the green score was performed through the AGREE (Analytical GREEnness Metric Approach and Software) tool developed by Pena-Pereira et al.^83^ In Figure 6, the result is shown with a score of 0.81 (closer to 1, the greener is the methodology), thus corroborating with the green potential of the technique. This score takes into consideration the 12 principles of green analytical chemistry^89,90^ and is transformed into a unified scale from 0 to 1, where the final score is calculated based on the principles of significance and presented in the form of a pictogram indicating the performance of the analytical procedure in each criterion and the weights assigned by the user.

**Figure 6:**
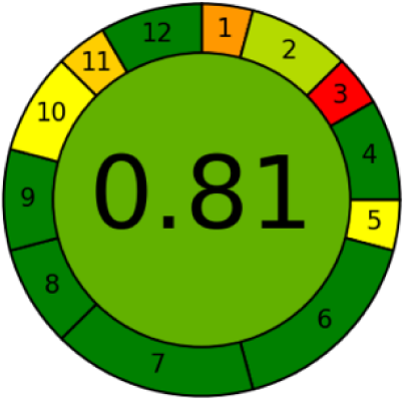
AGREE analysis.

Although several studies have brought natural products, such as biosorbents, for analytical purposes,^8–15^ none of them have physical characterization—chemical and morphological. Thus, this study demonstrated that RH is the best biosorbent considering all biomaterials and tested parameters, showing it as a promising biosorbent for analytical purposes. In addition, RH showed similar results when compared to C18, a commercial sorbent, corroborating its replacement by a natural sorbent and showing an excellent option for green chemistry techniques.

## 5 Conclusion

This study presents the most complete physicochemical and morphological characterization of natural sorbents discussed in the scientific literature, besides evaluating and selecting the best biosorbent used in the cleaning phase for the analysis of antipsychotics and anticonvulsants tested in HBM samples.

Morphological characterization showed that RH has even a relevant unevenness in its shape. This imperfection together with the nonroutine results of ZP, electrophoretic mobility, and particle size obtained during the physicochemical characterization proves the promising use of RH as a biosorbent for analytical purposes, especially when compared to C18, widely used commercially, obtaining statistically similar results between both.

The use of RH as a natural sorbent in the cleaning phase of the μQuEChERS technique for HBM samples showed successful results since the analytical response is already satisfactory, especially for the analytes found in large quantities in HBM such as CBZ. Therefore, RH is suitable for analytical purposes even if it makes the technique a green chemical procedure. Thus, additional studies should be carried out using RH as a biosorbent for the μQuEChERS technique for different biological matrices and other drugs and agricultural products collaborating with medical, pharmaceutical, and environmental research.

## Supplementary Material



## Notes

SEM Images of Commercial Sorbents (PSA, C18, and GCB) and RH sample in 5000× increase (Figure S1); Comparison of μQuEChERS Chromatograms of blanks, standards, and samples with Commercial Sorbents and Biosorbents (Figure S2) (PDF)
