# Supplementary material for: Use of Rice Husk as a Biosorbent for Analytical Purposes: Physicochemical, Morphological Characterization and Application in Human Breast Milk
Source: ACS Omega. 2025 Jun 6;10(23):24200–13. doi: 10.1021/acsomega.4c11200 (PMC12177769; doi:10.1021/acsomega.4c11200)
Supplement: Supplementary file 1 [file ao4c11200_si_001.pdf]

Use of rice husk as a biosorbent for analytical purposes: physicochemical, morphological characterization and application in human breast milk

Leonardo Corrêa Cardoso[1,2*]; Gustavo Andrade Ugalde[1,2,3]; Tielle Moraes de Almeida[2,4,5]; Jaquelini da Silva Reis[1]; Maria Odete da Silva Dalan[1]; Fernanda Ziegler Reginato[1,2]; Berenice de Oliveira Cruz Rodrigues[6]; Cristiane de Bona da Silva[2,4]; Fábio Andrei Duarte[7]; Ederson Rossi Abaide[8]; Marcio Antonio Mazutti[8]; André Valle de Bairros[1,2]

[1]Núcleo Aplicado a Toxicologia (NAT), Departamento de Análises Clínicas e Toxicológicas, Centro de Ciências da Saúde, Universidade Federal de Santa Maria, Santa Maria, Brazil.

[2]Programa de Pós-Graduação em Ciências Farmacêuticas, Centro de Ciências da Saúde, Universidade Federal de Santa Maria, Santa Maria, Brazil.

[3]Laboratório Integrado de Manejo de Pragas (LabMIP), Departamento de Defesa Fitossanitária, Centro de Ciências Rurais, Universidade Federal de Santa Maria, Santa Maria, Brazil.

[4]Laboratório de Pesquisa em Nanotecnologia e Desenvolvimento Farmacotécnico (NDF+), Departamento de Farmácia Industrial, Centro de Ciências da Saúde, Universidade Federal de Santa Maria, Santa Maria, Brazil.

[5]Laboratório de Superfícies e Macromoléculas (SMLab), Departamento de Física, Centro de Ciências Naturais e Exatas, Universidade Federal de Santa Maria, Santa Maria, Brazil.

[6]Unidade Obstétrica, Hospital Universitário de Santa Maria, Universidade Federal de Santa Maria, Santa Maria, Brazil.

[7]Programa de Pós-Graduação em Química, Departamento de Química, Centro de Ciências Naturais e Exatas, Universidade Federal de Santa Maria, Santa Maria, Brazil.

[8]Programa de Pós-Graduação em Engenharia Química, Centro de Tecnologia, Universidade Federal de Santa Maria, Santa Maria, Brazil.

---

[*]Corresponding author at: Leonardo Corrêa Cardoso

Núcleo Aplicado à Toxicologia, Centro de Ciências da Saúde, Prédio 26A, Sala 1401B, Universidade Federal de Santa Maria (UFSM), Santa Maria, 97105-900, RS, Brazil.

Email address: leonardo.cardoso@ufsm.br

Phone number: +55 55 98118-2701

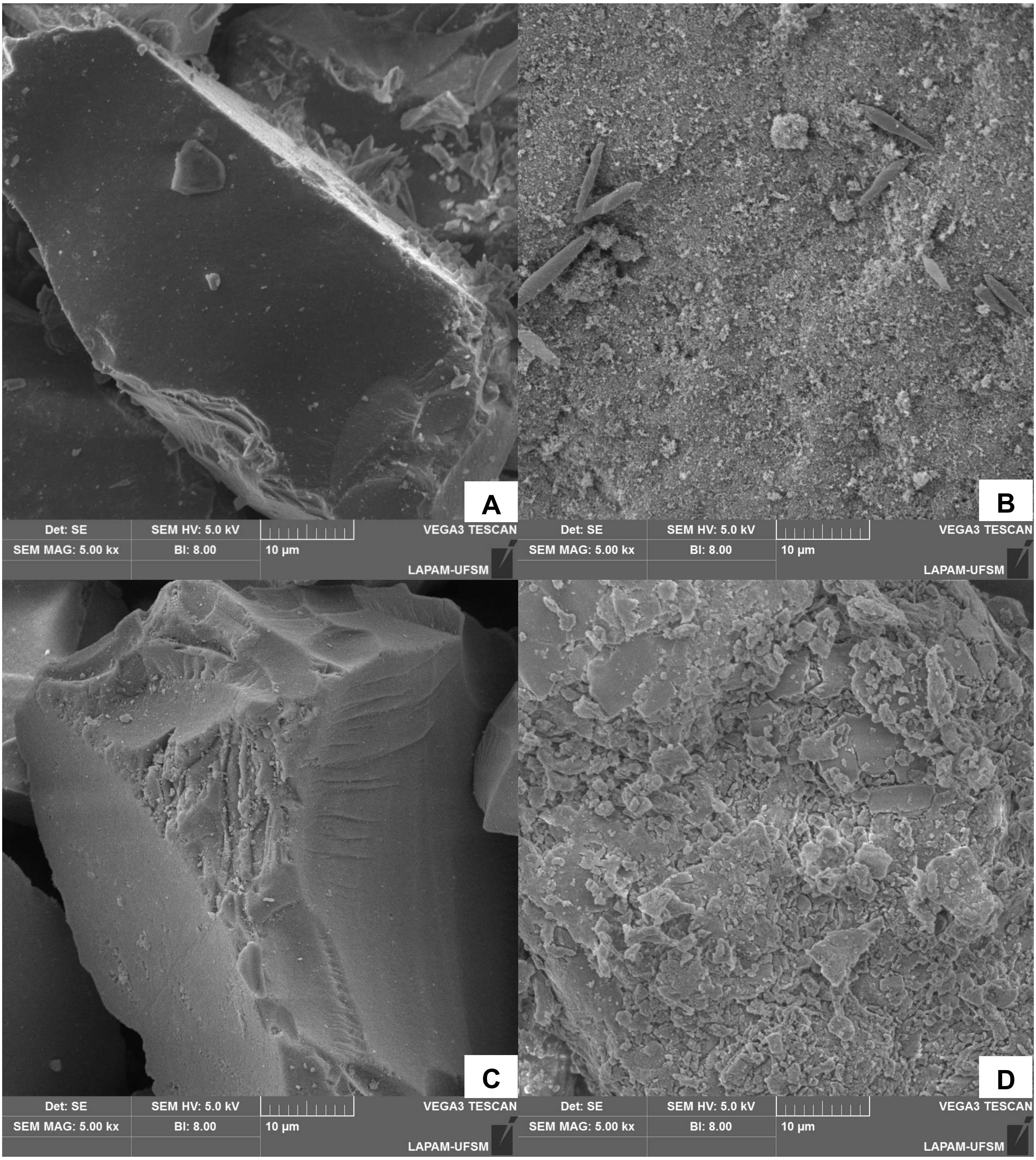

S1 - SEM images of commercial sorbents (PSA, C18 and GCB) and RH sample in 5000x increase.

Legend:
A: C_{18} ; B: GCB; C: PSA; D: RH.

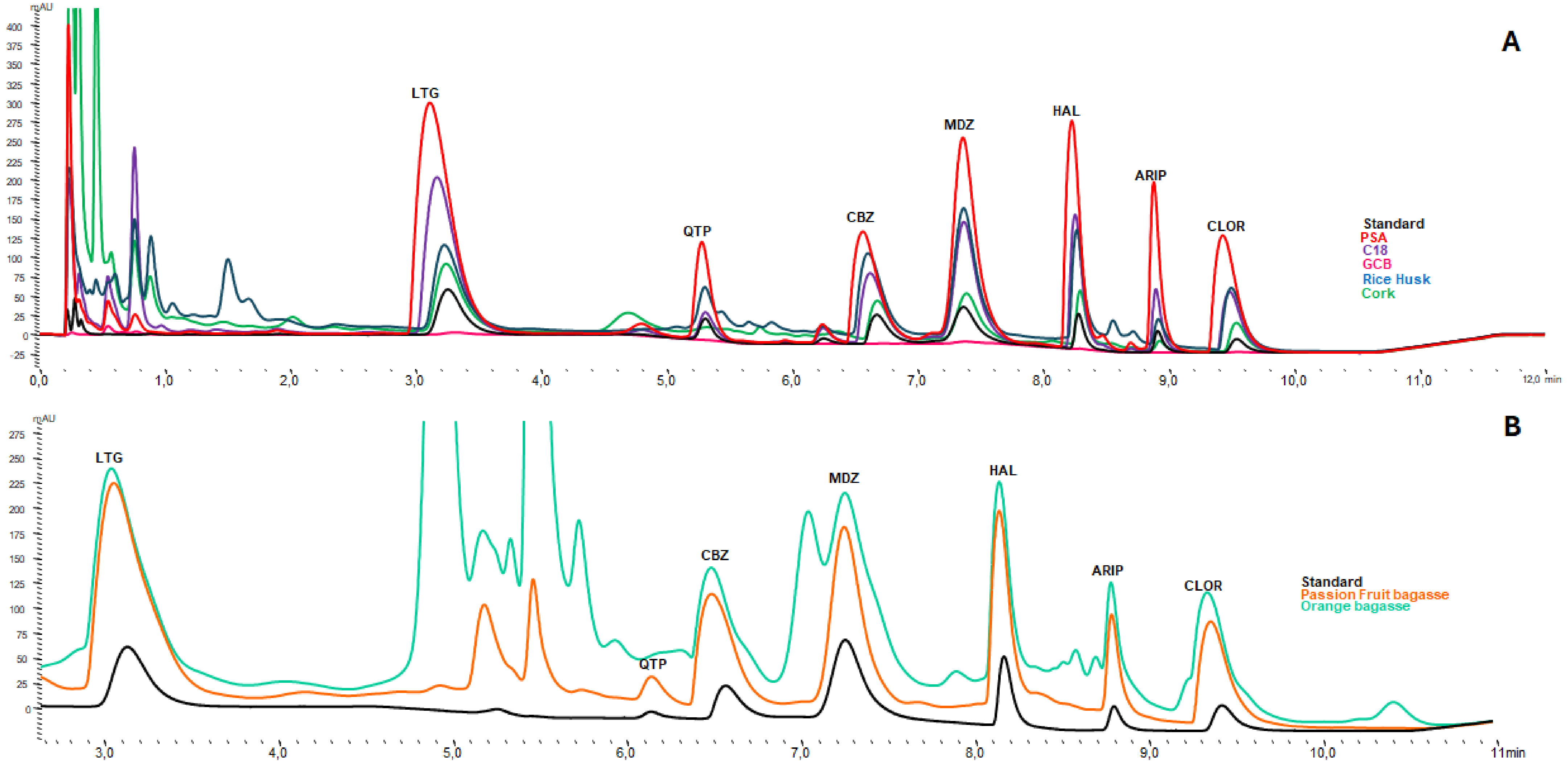

S2 - Comparison of µQuEChERS chromatograms of blanks, standards and samples with commercial sorbents and biosorbents.

Legend:

A: µQuEChERS chromatogram with commercial sorbents other than RH and Cork (biosorbents) with a standard; B: µQuEChERS chromatogram with biosorbents (Orange and Passion Fruit bagasse) with blank; Lamotrigine (LTG), Quetiapine (QTP), Carbamazepine (CBZ), Medazepam (MDZ), Haloperidol (HAL), Aripiprazole (ARIP) and Chlorpromazine (CLOR).
